# Insights from a Wolfram syndrome cohort: clinical and molecular findings from a specialized diabetes reference center

**DOI:** 10.20945/2359-4292-2024-0091

**Published:** 2024-09-24

**Authors:** Carolina Paniago Lopes, Gentil Ferreira Gonçalves, Maria Fernanda Vanti Macedo Paulino, Adriana Mangue Esquiaveto-Aun, Maricilda Palandi de Mello, Elizabeth João Pavin, Ikaro Soares Santos Breder, Mariana Zorron Mei Hsia Pu, Sofia Helena Valente de Lemos-Marini, Gil Guerra

**Affiliations:** 1 Universidade Estadual de Campinas Faculdade de Ciências Médicas e Hospital de Clínicas Departamento de Pediatria Campinas SP Brasil Departamento de Pediatria, Faculdade de Ciências Médicas e Hospital de Clínicas, Universidade Estadual de Campinas, Campinas, SP, Brasil; 2 Universidade Estadual de Campinas Laboratório de Genética Molecular Humana do Centro de Biologia Molecular e Engenharia Genética Campinas SP Brasil Laboratório de Genética Molecular Humana do Centro de Biologia Molecular e Engenharia Genética, Universidade Estadual de Campinas, Campinas, SP, Brasil; 3 Universidade Estadual de Campinas Faculdade de Ciências Médicas e Hospital de Clínicas Departamento de Clínica Médica Campinas SP Brasil Departamento de Clínica Médica, Faculdade de Ciências Médicas e Hospital de Clínicas, Universidade Estadual de Campinas, Campinas, SP, Brasil

**Keywords:** Wolfram syndrome, wolframin, WFS1, CISD2, diabetes mellitus, diabetes insipidus

## Abstract

**Objective:**

Considering the rarity and clinical and molecular diversity of Wolfram syndrome (WS), the objective of this study was to identify patients with a clinical presentation suggestive of WS following up at a single Brazilian diabetes service and analyze their clinical and molecular characteristics.

**Subjects and methods:**

The study included all patients with a clinical presentation of WS following up between 1991 and 2022 with early-onset diabetes mellitus and other WS signs and symptoms. A retrospective analysis was conducted, including patients’ age, sex, consanguinity, age at symptom onset, diagnosis of diabetes mellitus, optic atrophy, diabetes insipidus, neurological and psychiatric disorders, hearing loss, urinary disorders, hypogonadism, and *WFS1* molecular analysis.

**Results:**

Eight patients were identified, all of whom were diagnosed with diabetes mellitus at an average age of 3.7 years. Optic atrophy, diabetes insipidus, and hearing loss were common, while psychiatric and neurological alterations were observed in some cases. Genetic analysis revealed pathogenic variants in homozygosity or compound heterozygosity. The most frequent variant was p. Val412Serfs29, present in five of the seven families.

**Conclusions:**

This study represents the second-largest Brazilian sample of WS and is the first cohort from a single center in Southeast Brazil. The patients had an early, severe, and complete clinical presentation. The genetic variants identified were consistent with previous literature descriptions. The variant p. Val412Serfs29 was particularly common in this cohort, highlighting its relevance in the region.

## INTRODUCTION

Wolfram syndrome (WS) is an ultra-rare neurodegenerative disease characterized by a set of clinical manifestations also identified by the acronym DIDMOAD (DI for diabetes insipidus, DM for diabetes mellitus, OA for optic atrophy, and D for deafness) (1-4). The syndrome was first described in 1938 by Wolfram and Wagener in four of eight siblings with diabetes mellitus and optic atrophy (5). The initial and essential criteria for the diagnosis of WS are early-onset diabetes mellitus and optic atrophy (6). These criteria have a positive predictive value of 83% and a negative predictive value of 1% in diagnosing WS (1). A significant number of patients also develop psychiatric, endocrine, and urinary tract disorders. More than half of them develop significant neurological and cognitive deficits, which are recognized as the main presentations of this neurodegenerative disease (7).

The prevalence of WS has been estimated at 1:100,000 in North America (1) and 1:770,000 in the United Kingdom (8). However, these epidemiological studies were conducted in the pre-molecular era, before the *WFS1* gene was identified and the syndrome's various manifestations became more widely recognized. Using early-onset diabetes mellitus and optic atrophy as the minimum verification criteria for WS diagnosis, the estimated prevalence has been reported as 1:710,000 in Japan (9). Recent epidemiological data based on molecular typing of *WFS1* and confirmed by molecular diagnostic reports estimated the prevalence of WS to be 1:54,478 in the Messina district of Northeast Sicily (Italy) (10), 1:1,351,000 in Italy as a whole (11), and 1:805,000 in northern India (12).

Based on underlying genetic defects and clinical presentation, WS has been classified into type 1 (WS-1; Online Mendelian Inheritance in Man [OMIM] entry #222300) and type 2 (WS-2; OMIM #604928). Between both, WS-1 is undoubtedly the most prevalent type. Most patients have pathogenic variants in the *WFS1* gene inherited in a recessive pattern (4p16, OMIM #606201, 8 exons), which encodes the endoplasmic reticulum transmembrane protein wolframin (890 amino acids) (2,13,14).

Only a few patients with WS-2 have been reported worldwide; these patients carry recessive mutations in the *CISD2* gene (4q24, OMIM #604928), which encodes a small intramembrane protein of the endoplasmic reticulum known as CDGSH iron-sulfur domain-containing protein 2 (CISD2) (15-18). Patients with WS-2 share some of the clinical features observed in those with WS-1, including the development of diabetes mellitus, optic atrophy, and neurosensory deafness in the first two decades of life (15). However, WS-2 typically occurs with peptic ulcer and hemorrhagic predisposition but not with diabetes insipidus.

Advancement toward a disease classification based on molecular confirmation of the pathogenic variant (*WFS1* or *CISD2*) and the mode of inheritance (dominant or recessive) is currently necessary.

The survival of patients with WS is short. About 65% of these patients die before the age of 30-40 years, with an average age at death of 30 years (range 25-49 years) (11).

Considering the rarity of WS and its clinical and molecular diversity, this study aimed to identify patients with suggestive WS presentation following up at the Pediatric and Endocrinology Diabetes Outpatient Clinics at a single Brazilian diabetes reference center and analyze the clinical and molecular data of these cases.

## SUBJECTS AND METHODS

The study included all suspected cases of WS following up at a diabetes reference center in Southeast Brazil from 1991 to 2022. The inclusion criteria were patients with early-onset diabetes mellitus associated with at least one of the following conditions: optic atrophy, deafness, diabetes insipidus, or a neurologic, urinary, or psychiatric disorder. Most cases presented clinical symptoms of early-onset diabetes mellitus associated with optic atrophy, with or without other suggestive signs and symptoms of WS, as suggested by Urano (3). Until this article was finalized, two cases without optic atrophy had been identified, both in patients with early-onset diabetes mellitus. One of these cases was the sibling of a patient with a complete clinical presentation of WS, while the other had persistent polyuria despite maintaining optimal glycemic control.

A retrospective analysis of the medical records of each of these cases was performed, including current age (in years), sex (male or female), consanguinity between parents, age at symptom onset (in years), reason for the first consultation, age at diagnosis of diabetes mellitus (and whether ketoacidosis was present), and presence of optic atrophy, diabetes insipidus, neurological disorder, psychiatric disorder, hearing loss, urinary disorder, and hypogonadism.

All patients were followed up in the diabetes services of the same institution and evaluated by a trained ophthalmologist when necessary. The diagnosis of diabetes insipidus was established based on symptoms, laboratory results, and response to nasal desmopressin. Water restriction tests were performed when necessary. Patients with any related symptoms were referred for investigation with a neurologist, psychiatrist, otorhinolaryngologist, or urologist, if necessary.

Molecular analysis was performed using two different methods:

- Until 2019, the *WFS1* gene sequencing method was used. Following DNA extraction from peripheral blood, exons 2 to 8, non-coding exon 1, exon-intron junctions, as well as the 5’ promoter and 3’ UTR regions of the *WFS1* gene were amplified by polymerase chain reaction (PCR). Possible pathogenic variants were investigated with Sanger sequencing of the PCR products using the ABI PRISM Big Dye Terminator v3.1 Cycle Sequencing Kit (ABI PRISM/PE Biosystems, Foster City, CA, USA) and the Applied Biosystems 3500xL Genetic Analyzer (ABI PRISM/PE Biosystems). The freely accessible software programs 4Peaks (for iOS) and CLC Sequence Viewer v.7.6 were used for reading and comparing reference sequences NM_006005.3 and ENSG00000109501 for cDNA and gDNA, respectively. The identified variant was checked to verify registration in the Human Gene Mutation Database (https://www.hgmd.cf.ac.uk/ac/index.php) and SNP Database of the National Center for Biotechnology Information (https://www.ncbi.nlm.nih.gov/snp) under their respective access codes. The variant was classified according to the criteria by the American College of Medical Genetics and ClinVar (data obtained from Varsome, https://varsome.com).
- Starting in 2020, our hospital became part of the Rare Disease Services group of the Ministry of Health and the Rare Genomes Project, a partnership between the Ministry of Health and the Albert Einstein Israelite Hospital through PROADI-SUS. The patients’ DNA was extracted from peripheral blood and subjected to second-generation sequencing on the Illumina platform, after mechanical fragmentation and a PCR-free protocol. The data were processed for the detection of point variants, copy number variations, and structural variants following best practices for bioinformatics pipelines. The quality parameters for analysis were a minimum average coverage of 20 times for bases and a minimum depth of 90%. Variant numbering was performed using the reference transcript from the A base of the ATG initiation codon aligned against the GRCh38/hg38 reference genome. Variant classification was performed according to the criteria of the American College of Medical Genetics and following the updates of the ClinGen work group.

The statistical analysis included calculations of mean, median, minimum, maximum, and standard deviation values, and the resulting data are presented in Tables.

## RESULTS

Out of 14 patients with clinical presentation suggestive of WS, two were excluded due to death, two were lost to follow-up before molecular analysis, and two showed no molecular variants in the *WFS1* gene.

Table 1 shows the clinical characteristics of the eight patients with bi-allelic pathogenic variants in the *WFS1* gene. All patients presented with diabetes mellitus at an average age of 3.7 years (auto-antibodies were not evaluated in any of the cases). There was a predominance of the male over the female sex. Among the seven families, three had a history of parental consanguinity (first-degree cousins). Diabetic ketoacidosis was reported in half of the cases, and diabetes mellitus was well controlled with insulin. Optic atrophy was diagnosed in six of the eight patients at an average age of 10.1 years. Case 5B was included despite not having optic atrophy, diabetes insipidus, or deafness, as he had early-onset diabetes mellitus and was the sibling of a patient with a complete clinical presentation of WS. Case 7 was added as he had a persistent history of polyuria since the age of 4 years, despite optimal glycemic control. Given the patient's young age, a water restriction test was performed at the age of 9 years confirming the diagnosis of diabetes insipidus. In all, diabetes insipidus was diagnosed in five of the eight patients at an average age of 13.6 years at diagnosis. Notably, one of the patients without diabetes insipidus was 13 years old. Diabetes insipidus was well controlled with the use of nasal desmopressin. Deafness was diagnosed in five of the eight cases at an average age of 16.2 years. Only two patients (who are siblings, ages 17 and 13 years) and Case 6 (age 22 years) did not have deafness. All patients except for Cases 6 and 7 had psychiatric disorders, and four of the eight patients had neurological disorders. Urinary disorders were observed in four of the eight cases, and hypogonadotropic hypogonadism was present in only one case, a patient who was receiving replacement therapy with injectable testosterone.

**Table 1:** Clinical characteristics of eight cases with molecular-confirmed Wolfram syndrome

Clinical Data	Case 1	Case 2	Case 3	Case 4	Case 5A	Case 5B	Case 6	Case 7	%	Mean (SD) – (Minimum-Maximum)
Current age (years)	23	33	32	23	17	13	22	20		22.8 (76.8) – (13.0-33.0)
Diabetes mellitus (years)*	4	2	1	6	4	2	9	2	100	3.7 (2.6) – (1.0-9.0)
Optic atrophy (years)*	10	9	11	7	9	-	15	-	75	10.1 (2.7) – (7.0-15.0)
Diabetes insipidus (years)*	10	19	14	-	16	-	-	9	62.5	13.6 (3.7) – (9.0-19.0)
Hearing loss (years)*	21	10	18	17	-	-	-	15	62.5	16.2 (4.0) – (10.0-21.0)
Sex	M	F	F	M	F	M	M	M	62.5 (M)	
Consanguinity	-	-	-	+	+	+	+	-	50	
Initial presentation	DM	DM	DM	DM	DM	DM	DM	DM	100	
Diabetic ketoacidosis**	+	+	-	-	+	-	-	+	50	
Neurological disorder	PN	PN+E	PN	PN+E	-	-	-	-	50	
Psychiatric disorder	AXD	AXD+DT	DT+AD	AXD+AD	AXD	MA+AD	-	-	75	
Urinary disorder	-	NB	-	NB	NB	-	-	NB+PCD	50	
Hypogonadism***	-	-	-	+	-	-	-	-	12.5	

- = absent:

+ = present:

* = age at onset of symptoms:

** = at first presentation:

*** = hypogonadotropic hypogonadism: 5A and 5B = siblings. Abbreviations:

AD, attention deficit; AXD, anxiety disorder; DM, diabetes mellitus; DT, depressive disorder; E, epilepsy; F, female; M, male; MA, mood alteration; NB, neurogenic bladder; PCD, pyelocaliceal dilatation; PN, peripheral neuropathy; SD, standard deviation.

**Figure 1:**
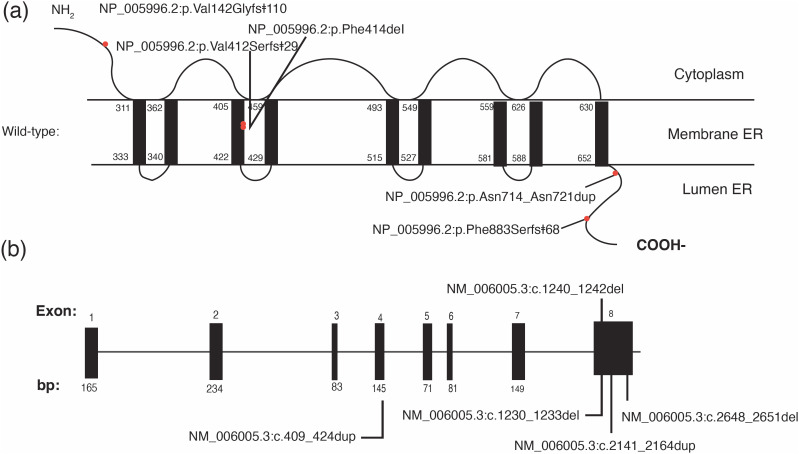
Schematic representation of the location of pathogenic variants (a) in the wolframin protein and (b) in the WFS1 gene. ER = endoplasmic reticulum.

Table 2 and Figure 1 show the results of the molecular analysis of the eight patients. All patients had pathogenic variants in either homozygosity (Cases 4, 5A, 5B, and 6) or compound heterozygosity (Cases 1, 2, 3, and 7). The identified variants were all located in exon 8, except for the variant in Case 4 and one variant in Case 7, which were located in exon 4. Among the six identified variants, p. Val412Serfs*29 was observed in five of the seven families and was the most recurrent, along with p. Val142Glyfs*110.

**Table 2:** Results of molecular analysis of eight patients with Wolfram syndrome

Case	Variant 1	Exon	Variant 2	Exon
1	c.2648_2651del	8	c.1230_1233del	8
	p. Phe883Serfs*68		p. Val412Serfs*29	
2	c.1240_1242del	8	c.1230_1233del	8
	p. Phe414del		p. Val412Serfs*29	
3	c.2141_2164dup	8	c.1230_1233del	8
	p. Asn714_Asn721dup		p. Val412Serfs*29	
4	c.409_424dup			4
	p. Val142Glyfs*110 (homozygosity)			
5A	c.1230_1233del			8
	p. Val412Serfs*29 (homozygosity)			
5B	c.1230_1233del			8
	p. Val412Serfs*29 (homozygosity)			
6	c.1230_1233del			8
	p. Val412Serfs*29 (homozygosity)			
7	c.409_424dup	4	c.1243_1245del	8
	p. Val142Glyfs*110		p. Val415del	

## DISCUSSION

With autosomal recessive inheritance, WS-1 is an ultra-rare, progressive, neurodegenerative disease. The minimal diagnostic criteria for WS-1 include the association of non-autoimmune diabetes mellitus and optic atrophy, often accompanied by other findings such as diabetes insipidus, deafness, and psychiatric and neurological disorders (1-6).

The present study represents the second-largest case series of patients with WS in Brazil and the first case series from a single center in Southeast Brazil. The largest Brazilian study of this kind was published by Gasparin and cols. in 2009 and included 27 patients from 19 unrelated families across all Brazilian regions (19).

In the present study, all WS cases had early-onset diabetes mellitus (average age at onset 3.7 years). In a large English cohort of 45 patients and in the Brazilian cohort (19), the average age of the patients at diabetes mellitus onset was 6 years.

In the present study, optic atrophy was diagnosed in six of the eight cases at an average age of 10.1 years, which is comparable to the findings from the English (1) and Brazilian cohorts (19). In a study by Heredia and cols., 85% of patients had developed both diabetes mellitus and optic atrophy by the age of 18 years; therefore, these two criteria should not be used as absolute requirements for WS diagnosis, indicating that the clinical manifestations in WS are widely heterogeneous (20). Ophthalmic manifestations of WS are variable, but most patients present with other neuro-ophthalmological findings beyond optic atrophy. Over 80% of patients with WS have decreased central visual acuity, color vision deficits, visual field defects, retinal nerve fiber layer (RNFL) thinning, and optic disc pallor (21). Other neuro-ophthalmological findings may include a large cup-to-disc ratio, nystagmus, and strabismus (21). Patients with RNFL thinning, strabismus, and nystagmus tend to have more severe systemic disease (21). Therefore, patients with suspected WS should periodically undergo careful examination by a neuro-ophthalmologist.

In five cases in the present study, the onset of diabetes insipidus occurred at an average age of 13.6 years, while in the English cohort (1), the average age at onset was 14 years. Also in the present study, deafness was diagnosed in five cases at an average age of 16.2 years, which is comparable to findings from the English cohort (1). Thus, the cases in the present study exhibited clinical characteristics similar to those of the English (1) and Brazilian (19) cohorts.

Despite being an ultra-rare autosomal recessive disease, consanguinity was present in only three of the seven (42.8%) families in the present study, which is consistent with the corresponding finding from the Brazilian cohort (19).

Regarding molecular aspects of the cases in the present study, all the identified variants were located in exon 8 of the *WFS1* gene, except for two cases, which were located in exon 4. This pattern is consistent with findings from other studies, as exon 8 is the largest exon and encodes the nine transmembrane domains of the protein. The p. Val412Serfs*29 variant was the most common variant; it was observed in five of seven families in the present study and was also prevalent in the Brazilian cohort (19).

The p. Val412Serfs*29 variant has been described in international studies but at a much lower frequency than that found in Brazil. Other variants found in the present study, such as p. Phe884Serfs*68 and p. Val142Glyfs*110, have been reported by others (22-25). Two additional variants identified in the present study – p. Asn714_Asn721dup and p. Phe414del – are less commonly reported (2,24).

The inheritance pattern of *WFS1* gene variants can affect the onset and severity of WS clinical features. Patients with two inactivating variants may experience an earlier onset of diabetes mellitus and optic atrophy, while compound heterozygotes for missense variants may exhibit a milder phenotype (24,26). Complete loss-of-function variants seem to lead to early-onset diabetes mellitus (11,27). The variants identified in the present study were all complete loss-of-function mutations, explaining the comprehensive and early clinical presentation. Multiple studies have elucidated genotype-phenotype correlations in WS, which are an important tool for the clinician as they help predict the age of onset and severity of the different clinical features. It was determined that the presence of two inactivating variants (nonsense or frameshift) – as observed in all patients in the present study – predisposed to an earlier age of onset for both diabetes mellitus and optic atrophy (28).

In Brazilian patients, the presence of variants described worldwide is attributed to the extensive population mixing due to historical migration movements. After Brazil was discovered in the 16th century, Portuguese settlers arrived, followed by substantial African slave trafficking. In the 19th century, European immigrants, particularly Italians, Portuguese, Spaniards, and Germans, contributed to the diversity of the population. The 20th century experienced an influx of Asian immigrants, including Japanese and Syrian-Lebanese ones. These great immigration waves have resulted in a genetically diverse Brazilian population (29).

In conclusion, this study presents the second-largest Brazilian case series of WS, the first from a single center exclusive to the southeastern region of the country. The patients had a severe, complete, and early clinical presentation, which can be explained by the severity of the *WFS1* gene variants observed. Six different variants were identified, all of which have been previously described. The p. Val412Serfs*29 variant showed a frequency of 72.4%, notably higher than the other Brazilian cohort (47.4%) and international studies (6.8%).
